# Implications of human activities for (re)emerging infectious diseases, including COVID-19

**DOI:** 10.1186/s40101-020-00239-5

**Published:** 2020-09-25

**Authors:** Nundu Sabiti Sabin, Akintije Simba Calliope, Shirley Victoria Simpson, Hiroaki Arima, Hiromu Ito, Takayuki Nishimura, Taro Yamamoto

**Affiliations:** 1 Department of International Health and Medical Anthropology, Institute of Tropical Medicine, Nagasaki University, 1-12-4 Sakamoto, Nagasaki, 852-8523 Japan; 2 Graduate School of Biomedical Sciences, Nagasaki University, Nagasaki, Japan; 3 Leading Program, Graduate School of Biomedical Sciences, Nagasaki University, Nagasaki, Japan; 4 Department of Human Science, Faculty of Design, Kyushu University, Fukuoka, Japan; 5 Department of Public Health, Graduate School of Biomedical Sciences, Nagasaki University, Nagasaki, Japan

**Keywords:** Humans, Factors, (Re)emerging infectious disease, Coronavirus

## Abstract

Since 1980, the world has been threatened by different waves of emerging disease epidemics. In the twenty-first century, these diseases have become an increasing global concern because of their health and economic impacts in both developed and resource-constrained countries. It is difficult to stop the occurrence of new pathogens in the future due to the interconnection among humans, animals, and the environment. However, it is possible to face a new disease or to reduce the risk of its spread by implementing better early warning systems and effective disease control and prevention, e.g., effective global surveillance, development of technology for better diagnostics, effective treatments, and vaccines, the global political will to respond to any threats and multidisciplinary collaboration involving all sectors in charge of good health maintenance. In this review, we generally describe some factors related to human activities and show how they can play a role in the transmission and spread of infectious diseases by using some diseases as examples. Additionally, we describe and discuss major factors that are facilitating the spread of the new pandemic known as COVID-19 worldwide.

## Background

Emerging infectious diseases (EIDs) are diseases recently identified or previously unknown infections that cause public health threats, either locally or internationally [1]. In contrast, reemerging infectious diseases are known diseases that reappear and increase in incidence but that in the past may have fallen to levels so low that they were no longer considered a public health problem [1]. In the twenty-first century, these diseases have increasingly become a global concern [2] due to their health and economic impacts in both developed and resource-constrained countries. It has been estimated that out of approximately 400 known emerging pathogens, 25% occur as human pathogens [3]. In other reports, approximately 175 species are associated with emerging diseases, of which approximately 75% are of zoonotic origin [4]. Most of these zoonoses have occurred due to increased contact between humans and animals [5–8]. These zoonoses account for approximately 44%, 30%, 11%, 9%, and 6% of viruses or prions, bacteria or rickettsia, protozoa, fungi, and helminths, respectively [9]. In the post-1980 period, some zoonotic pathogens were responsible for outbreaks such as *Escherichia coli* O157 in the 1980s [10], the avian strain of influenza in Hong-Kong in 1997 [11], West Nile virus in 1999 [12], the outbreak of chikungunya fever in Italy in 2007 [13], the epidemic of Q fever in the Netherlands [14, 15], the outbreak of severe acute respiratory syndrome (SARS) in 2002 caused by very virulent strains of new coronaviruses [16–18], Middle East respiratory syndrome coronavirus (MERS-CoV) in 2012 [19], and avian influenza, such as A/H1N1 in 1997 in Hong-Kong [20], A/H7N7 in 2004 in the US [21], A/H7N2 in 2007 in the UK [22], and A/H7N9 in China [20, 23]. Recently, major outbreaks of Ebola virus disease (EVD) threatened populations in West African countries between 2013 and 2016, with 28,603 cases and 11,310 deaths [24], and in the Democratic Republic of Congo (DRC) from 2018 to 2020, with 3444 cases and 2264 deaths [25]. Currently, the entire world is facing an unprecedented pandemic caused by a new strain of coronavirus called coronavirus disease 2019 (COVID-19) [26].

Wolfe et al. [27] identified five stages leading to endemic human disease, where a pathogen restricted to infecting animals may be transformed into a human pathogen. The first stage is when a pathogen is present in animals but has not yet been detected in humans under natural conditions; the second is when a pathogen has been transmitted from animals to humans, but there is no evidence of human-human transmission (e.g., tetanus, rabies); the third is when a pathogen can undergo only a few cycles of secondary transmission between humans and shortly die out, where a subsequent wave will require the involvement of animal hosts (e.g., Ebola virus, Marburg virus, monkeypox virus); the fourth is when a pathogen undergoes long sequences of secondary transmission between humans without the involvement of animal hosts (many vector-borne diseases, e.g., dengue virus, yellow fever virus, cholera, influenza A, typhus); and the fifth is when a pathogen previously restricted to animals later evolves into an exclusively human pathogen (e.g., smallpox, measles, syphilis, falciparum malaria, rubella, mumps, hepatitis B, tuberculosis, AIDS) [27]. Consequently, the passage from one stage to another depends on the host-pathogen-environment interconnection [28]. The emergence of infection is influenced by the interconnecting domains of determinants such as physical and environmental factors; ecological factors; genetic and biological factors; and social, political, and economic factors [29]. Therefore, these determinants may influence the emergence and spread of infectious diseases. According to Chareonsook et al. [30], human demographic changes and behavior, travel and commerce, ecological changes, technology and industry, microbial adaptation and change, and the breakdown of public health measures are sufficient factors for the emergence and spread of infectious diseases. Subsequently, Lederberg et al. [29] identified additional factors such as human vulnerability, economic development, land use, lack of political will, poverty and social inequality, war and famine, and intent to harm.

In the twenty-first century, many outbreaks have occurred around the world, such as EVD, avian influenza, and coronaviruses (SARS, MERS, COVID-19), because of factors related to human activities. Here, we will describe some human-related factors and give some examples.

## Human-related factors influencing the transmission and spread of infectious diseases

### Globalization (international travel and trade)

The twenty-first century has ushered in an era where infectious diseases are occurring at an unprecedented frequency and speed. A coordinated global response is needed; however, in this globalized environment of interdependent trade, travel, and migrations, pathogens have no geopolitical borders [2, 31].

Recently, EIDs such as COVID-19, EVD, Lassa fever, and SARS have been an issue of global concern. With globalization, the risk of the emergence of new diseases is increasing since humans have more chances to be exposed to new pathogens. Additionally, the risk of international spread of infectious diseases is increasing because of increased international travel. The process of globalization potentially influences a broad range of biological, environmental, and social factors that affect the burden of many critical human infections [32]. Various international organizations and governments have been trying to establish better global systems to respond to EIDs. However, the SARS outbreak in 2003, the EVD outbreak in West Africa in 2014, and the current COVID-19 pandemic have highlighted that the current global system is still not fully ready for such (re)emerging infectious diseases [33]. These EIDs threaten public health, as well as the global economy, and are sustained by increasing global trade and travel. However, the disruption of the human-ecological system is another potential factor. Travelers could play a role in importing EIDs and could be a carrier of major epidemics [34]. It has been observed that (re)emerging infectious diseases are imported by travelers or tourists in Europe, such as chikungunya and dengue in France and Italy as well as COVID-19 in France from China [35–37]. Cross-border movements of people, as well as trade in goods and services, are increasing the challenges for infectious disease control. As an example, in 2005, evidence of outbreak importation through international travel was found with the isolation of H5N1 type A influenza virus from two mountain hawk eagles that were illegally imported from Thailand in an airline cabin carry-on baggage to Belgium [38]. The risk of infectious diseases rises with the increased mobility of people, growth in the international trade in food and biological products, and social and environmental changes. It is evident that trade and international travel are essential to humankind. However, some preventive measures, such as travel restrictions and creation of awareness among trade and travel stakeholders, are paramount to flatten the curve. In recent years, trade embargoes or travel restrictions have been replaced with sensitive early warning surveillance, rapid verification procedures, and international response networks as well as epidemic preparedness through stockpiles of essential medicines [39, 40]. However, studies have proven that if implemented within 2 weeks of cases, travel restrictions are 99% effective in conjunction with border restrictions that prevent the entry of infected travelers. Additionally, a 90% restriction on long-distance flights can lead to delays in the epidemic peak that range from a few days to a few weeks [41–44]. These developments affect all elements of the infectious disease chain: hosts, agents, and vectors [45]. Moreover, we must not ignore the fact that outbreaks are themselves associated with significant costs in terms of lost trade and tourism revenue. Therefore, a fear of economic penalties and social stigma can arise and can sometimes lead authorities to underreport data in epidemics, hence risking serious public health consequences.

### Changes in environment/ecological changes

Global environmental changes are due to human modifications to the environment [46]. National, regional, and global environmental threats to human health according to the World Health Organization (WHO) include “climate change, stratospheric ozone depletion, changes in ecosystems due to loss of biodiversity, changes in hydrological systems and the supplies of fresh water, land degradation, urbanization, and stresses on food-producing systems” [47].

Changes in the environment, including food-handling practices, agriculture, and changes in water ecosystems, seem to increase the chance of contact with a reservoir host encountering microorganisms previously unknown to humans. Anthropogenic changes alter the environment by increasing the risk of transmission of pathogens unfamiliar to humans [48].

It has been established that there is a relationship between infectious disease outbreaks and changes in the environment, including climate change events [49]. Ecological changes are one of the most important drivers of (re)emerging infectious diseases, specifically agricultural development, climate changes, food-handling practices, changes in water ecosystems, deforestation, and reforestation [48, 50, 51].

Climate change results in differences in weather conditions and patterns of extreme weather events. Spatiotemporal changes in climate affect the reproduction, development, survival, and livability of pathogens, hosts, and their interaction with human beings. Dramatic and unforeseen changes in weather conditions have profound effects on many infectious diseases. Extreme weather events and meteorological hazards make it more complicated to predict the implications for disease pathogens, hosts, and transmission [52–54]. The United Nations High Commissioner of Refugees (UNHCR) estimates that an average of 22.5 million people have been displaced by weather-related events or climate since 2008 [55]. The literature on climate variations and infectious diseases demonstrates that climate-sensitive diseases, i.e., vector-borne and food/water-borne diseases, are vastly affected by climate variability and climate change [56, 57]. Vector-borne diseases (VBDs) are the most climate sensitive. They have several climate drivers [58], causing over 700,000 deaths annually, accounting for more than 17% of all infectious diseases and having the highest burden in tropical and subtropical areas, particularly affecting the most deprived populations [59]. Some examples of VBDs of significance to humans and animals are malaria, schistosomiasis, dengue, chikungunya, and Lyme disease [60–62]. Improving infrastructure and disease surveillance systems worldwide and funding health observatories would help to better detect and attribute the effects of climate change on VBD (re)emergence [60–62]. The burden of food- and water-borne diseases (FWBDs) is found in some parts of developing countries and areas where food and water hygiene are inadequate. For example, in Africa, FWBDs account for 91 million illnesses, resulting in 137,000 deaths, annually [63]. Important causes of food-borne diseases are noroviruses, *Campylobacter* spp., and nontyphoidal *Salmonella*, while those of water-borne diseases remain diarrheal diseases, such as cholera, *Shigella*, and poliomyelitis. Additionally, food-handling practices have been associated with SARS and H5N1 outbreaks [48]. Proper water, sanitation, and hygiene interventions can significantly reduce FWBDs [63].

Agricultural development is a factor and the most typical way humans alter and interpose themselves into the environment [64, 65]. It is the world’s single most significant driver of global environmental change and is key to attaining Sustainable Development Goal (SDG) 2, which aims to end hunger, achieve food security and improved nutrition, and promote sustainable agriculture [66, 67]. The expansion and intensification of agriculture come with massive habitat conversion, increasing use of agricultural inputs such as antibiotic growth promoters and pesticides, and contamination with animal waste [68]. Several studies have linked agricultural land use or land use change with infectious disease risks. Significant associations were identified for malaria, spotted fever group rickettsioses, hookworm, scrub typhus, *Schistosoma japonicum*, and *Trichuris trichiura*.

On the other hand, agricultural practices were implicated in the pandemic influenza outbreak, Nipah, and West Nile transmission [48]. Exposure to livestock could result in 2–4 times the risk of infection with vector-borne, bacterial, or zoonotic diseases [69]. To withstand the future viability of agriculture, sustainable practices are required to meet rising human needs while contributing to the resilience and sustainability of landscapes, biospheres, and the Earth system [67]. It has also been shown that deforestation and reforestation can influence the emergence of some pathogens. For example, reforestation has been linked to Lyme disease outbreaks [56, 70].

### Human-wild animal interface

Approximately three-fourths of human EIDs are caused by zoonotic pathogens [71]. However, according to the WHO, humans coexist with animals in a variety of ways, either by hunting [72] or by living as domestic animals [73]. The interface among humans, animals, and the environment we share can be a source of diseases impacting public health [74]. However, in most cases, reported causes for and drivers of the apparent trends in disease emergence are lacking in intense evidence-based research. However, there is circumstantial evidence to suggest that novel epidemics and disease syndromes are the results of changing agroecology and human behavior and movements, intensifying interfaces with certain wildlife species and climate change [6, 75]. Although no clear evidence has been presented to date, hunting has been associated with zoonotic diseases [72]. With modern ways of life, however, this hypothesis became obsolete with the low spillover from wild animals to humans. The possible reason was that when people lived as hunter-gatherers, they were always on the move, and it became difficult for microbes to keep up with their human hosts. Once people started living as farmers, they began residing in more significant numbers in the same place and were in daily contact with their accumulating feces for extended periods [76]. Other evidence has shown that bushmeat is an essential source of protein and income in Africa despite its link with numerous EIDs, such as Ebola and Marburg [7]. The increased demand and commercialization of these types of meat have exposed people to pathogens and thus facilitated the geographic spread of the diseases [77]. All the aspects highlighted above have created conditions highly amenable for the spillover of microbes into the human ecosystem.

Moreover, the domestication of wild animals has narrowed the gap between wild animals and humans. Evidence has shown that domesticated animals that were long ago associated with humans are the ones at the center of those that favor parasite/pathogen transmission not only to humans but also to all other domesticated animals [78]. It has been revealed not only that humans are victims of the domestication of wild animals but also that the reverse is true. An example is that the occurrence of *Taenia* tapeworms in humans predated the development of agriculture, animal husbandry, and domestication of cattle or swine, and later, it accompanied early human dispersion out of Africa. Swine and cattle are thought to have acquired infections with *Taenia* species during their early domestication [79]. Thus far, it is not possible to stop human-animal interconnections. However, it is possible to reduce the negative impact of the human-wild animal interface, which contributes to the emergence of infectious diseases, by responding before the occurrence of spillover events, epidemics or even pandemics [80]. For example, people should be educated to stop harvesting bushmeat, especially by avoiding nonhuman primates; future zoonotic spillover events should be prevented through culturally appropriate education, and disease spread should be avoided through better disease surveillance [81, 82].

### Antimicrobial resistance

The resistance of microbes (viruses, bacteria, parasites, and fungi) to antimicrobials has contributed to the re-emergence of many diseases that were no longer considered a public health problem [83], narrowing the range of antimicrobial drugs and representing a global public health concern [84–86]. It has been estimated that approximately 700,000 annual deaths worldwide are a result of antimicrobial resistance (AMR) [87], which poses a problem to achieve the United Nations SDGs [88] and might reduce the gross domestic product by approximately 3.8% by 2050 [89].

It has been shown that AMR is the consequence of microbial adaptation and genetic changes that allow them to bypass the human immune system, infect human cells, and spread disease. These microbes can emerge and spread rapidly and become resistant to multiple antimicrobials at the same time [90–94] by developing mechanisms to exchange or incorporate new genetic material into their genomes, exchanging virtually any stretch of DNA or RNA [95]. Sometimes, even doctors prescribe inappropriate antimicrobials (i.e., an antimicrobial is not needed at all, or they prescribe the wrong drug or wrong dose) [96], or physicians prescribe drugs with the wrong prescription doses or antibiotics with an incorrect duration of treatment [97]. This practice contributes to the growth and spread of microbial resistance [98] and can be responsible for nosocomial infections in hospitals. For instance, more than 90% of *Staphylococcus aureus* species were resistant to penicillin and beta-lactam antibiotics in hospitals in the United States (US) and were responsible for approximately 19,000 deaths in 1992 [83]. Additionally, between 1989 and 1993, the incidence of vancomycin-resistant enterococci increased 20-fold in the US, and in 1997, the susceptibility of *Staphylococcus aureus* to vancomycin decreased in both the US and Japan [99]. In the European Union (EU), approximately 15–50% of *Klebsiella pneumoniae* species are resistant to carbapenems [100]. Previously, methicillin-resistant *Staphylococcus aureus* (MRSA) was associated with severe invasive disease only in hospitals; however, in the last 20 years, it has been identified in the community and named community-associated (CA)-MRSA [101]. As an example, CA-MRSA pneumonia is responsible for approximately 75–85% of admitted patients and 20 to 60% of deaths in the intensive care unit [102]. Currently, extended-spectrum β-lactamase resistance is endemic in hospitals and the community worldwide [103, 104].

Moreover, multidrug-resistant (MDR)-*Acinetobacter baumannii* isolates carrying Cefotaxime-M (CTX-M) enzymes are now responsible for nosocomial infections and among specific populations and accountable for high mortality and morbidity [105], as well as cholera O1 and O139 [106–109]. Additionally, it has been shown that the prevalence of MDR-TB among new TB cases increased from 12% in 2009 to 13.7% in 2010 in the EU. Thus, drug-resistant TB is still a major concern despite a decrease in TB incidence in subsequent years [110, 111].

The use of antimicrobials as additives in plant agriculture (fruits, vegetables, orchids, etc.) and animals and, more importantly, food-producing animals can lead to the development of resistant bacteria [112]. Thus, resistant genes in animals can be transferred to humans through the consumption of food or direct contact with food-producing animals or through environmental spread [113–122]. The misuse of antimicrobials, especially in developing countries, is mainly due to their unregulated supply chain, the availability of over-the-counter drugs [123–126], and self-medication [125, 127–131], which is amplified by poverty [131]. Up to half of all antibiotics prescribed are misused and neither needed nor effective [132, 133]. Another situation is the discontinuation of treatment by patients with a recurrent infection, which contributes to the development of a virulent and resistant strain of the microbe [130]. All these factors contribute to genetic selection pressure for the emergence of MDR bacterial infections in the community. Given the factors that contribute to the rise of AMR, reducing its risk implies the need to improve in ensuring the appropriate use of antibiotics through the enforcement and improvement of legislation on prescribing and dispensing antibiotics. In addition, the use of antibiotics in animal medicine and food production must be regulated. The government, pharmacists, doctors, veterinarians, and agronomists must work together and establish national guidelines for dispensing and prescribing antimicrobials. Adequate training on the proper prescription of antimicrobials must be given to health professionals, and the population must be educated on the use of antimicrobials and the threat of AMR.

### Host susceptibility

Human susceptibility may influence the emergence of infectious diseases through genetic polymorphisms, impaired immune function, malnutrition, AIDS, immunosuppressive conditions, aging, and others. Innate and acquired immune defenses help humans to fight against infectious diseases. Genetic polymorphisms are potent natural selective forces for pathogens to induce human defenses against infection [134–136]. People suffering from hemoglobinopathy S homozygosity develop more severe *Plasmodium falciparum* infection than those who have hemoglobin S heterozygotes [136, 137]. Malnutrition also increases the severity of some infectious diseases. Thus, malnourished children have a higher risk of death from diarrhea, acute respiratory infections, and possibly malaria [138]. According to the WHO, 26% of the world’s malnourished children have been affected by population displacements, natural disasters, wars, civil disturbances, and population growth in the region [139]. Considering immunosuppressive conditions, people who suffer from HIV/AIDS and cancers have a higher risk of increased disease severity. For example, the emergence of fungi, such as *Aspergillus* spp., and other opportunistic agents that were previously uncommon or unrecognized have increased in populations with cancer chemotherapy, organ, and tissue transplantation, or those infected with HIV [140]. It has also been shown that more than 10% of cancers or transplants are either colonized or infected with vancomycin-resistant enterococci [141].

### Behavior changes

Human behavior has been an essential factor in the transmission of emerging infectious pathogens. Understanding community behavior during epidemics is key to improving control strategies [142]. Behavior changes are often linked to culture, which refers to integrated patterns of human behavior that include the beliefs, language, communications, actions, and organizations of racial, ethnic, religious, or social groups [143]. Many infectious disease outbreaks sensibly spread because of these factors. Since the occurrence of HIV/AIDS and Ebola, for example, stigmatization, discrimination, ignorance, trust, and misinformation have been the root of the spread of these diseases [144–146].

Another situation concerns human behavior changes related to conflicts. Conflicts of human behavior occur where people are interconnected, and they result from a variety of attitudes, needs, beliefs, and ways that humans react to different situations [147]. Conflict situations can potentiate the (re)emergence and transmission of infectious diseases and are characterized by war or civil strife. This usually leads to the displacement of large populations, interruption of primary care and immunization programs, inadequate safe water supply, poor sanitation, and increased exposure to disease vectors [67–69]. According to UNHCR, 70.8 million people around the world are forcibly displaced, constituting 41.3 million internally displaced people, 25.9 million refugees, and 3.5 million asylum-seekers [70] from Africa to the Middle East, South Asia, and Europe [148, 149]. Millions of people live in deplorable conditions, and healthcare facilities in host countries are overburdened; these conditions are suitable for the (re)emergence of infectious diseases [70, 150]. Displaced populations are prone to viral and bacterial respiratory infections, nosocomial antibiotic-resistant bacterial infections, cholera, cutaneous leishmaniasis, sexually transmitted infections, poliomyelitis, Chagas, schistosomiasis, hepatitis B and C, meningococcal infections, and VBDs [57, 151–154]. It would be better to (1) strengthen health systems in all countries; (2) evaluate the detection, containment, treatment and control of infectious diseases, including the implementation of surveillance systems; and (3) devise strategies for epidemics and protect health-supporting infrastructure and health workers during conflict situations and the aftermath, ultimately striving to achieve health equity by key policy actors [68, 81, 151, 155, 156].

### Factors related to the spread of COVID-19

COVID-19 is caused by a virus officially named severe acute respiratory syndrome coronavirus-2 (SARS-Cov-2) [157, 158]. It is a new pandemic that started in December 2019 in Wuhan, Hubei Province of China [159–161]. The disease rapidly spread from Wuhan to the rest of Mainland China and other parts of the world, affecting at least 216 countries and territories, with 15,745,102 confirmed cases and 639,317 confirmed deaths as of 26 July 2020 [26, 162, 163]. Countries are reacting differently regarding the WHO-recommended preventive measures (lockdown, test-trace isolation, wearing masks and wearing them correctly, covering the mouth when coughing and sneezing, keeping at least one meter of social distance, frequent washing of hands and avoiding direct contact of the eyes, nose, and mouth [164]), making some countries more affected than others. Therefore, the variation in the severity, frequency, and amplitude of this pandemic is due to the addition of several factors as well as government policies.

Human behavior is an essential determinant of the transmission and spread of COVID-19 because the pandemic is spread by individual movements through large droplets and contacts, and possibly by aerosols and fomites, from person to person with direct or indirect contact based on the experience with SARS-CoV and MERS-CoV [165–167]. Self-protection may be the critical determinant for the prevention of COVID-19 [168] from one person to another because when a person protects himself or herself, he or she also protects others. Thus, understanding the collective behavior between and within individual groups would be useful for decision-making. Behavioral differences regarding COVID-19 containment measures such as lockdowns, social distancing, and the use of facial masks may cause some individuals to be more at risk than others and some countries to be more affected than others. Community behavior regarding preventive measures is influenced by cultural habits and misinformation. For example, there was also a great deal of misinformation and rumors that were widespread among the Chinese population and abroad with regard to the virus origin [169–172], transmission mode, treatment, prevention, and control measures [173, 174]. In addition, the WHO also created misinformation at the beginning of the pandemic by restricting the wearing of masks only to sick people showing symptoms and those who are caring for people with suspected infection [175]. Thus, some countries advised populations to wear masks only in enclosed spaces such as shops or public transport. Additionally, misinformation created from social media and websites with no credible evidence has generated changes in the behavior of many people, increasing the frequency and spread of COVID-19 locally, regionally, and internationally [176, 177]. In some countries, such as Japan, Singapore, China, and South Korea, cultural habits—such as the culture of mask-wearing, minimal personal contact, improved hand sanitization, and fanciful suggestions that Japanese speakers emit fewer potentially virus-laden droplets when talking than do speakers of other languages—have helped to flatten the curve and to limit the spread of COVID-19 in communities [178, 179]. On the other hand, in Western countries such as Italy, Spain, the United States, the United Kingdom, France, and many other countries where the culture of mask-wearing is not a habit and where hugging, kissing, shaking hands, spending time together, and engaging in other social practices are common, the virus has spread quickly [180, 181].

Another factor is the lack of transparency from government, and politicization of this outbreak by politicians has created confusion among communities and urged people not to follow prevention and control measures such as early detection, isolation, and treatment of cases, contact tracing, and containment measures in communities to prevent disease transmission within and between societies.

Host susceptibility includes aged individuals and other individuals with compromised immunity (such as high blood pressure, HIV, kidney diseases, diabetes, and cancers) who face higher risks of COVID-19 [182–184]. Immune-compromised individuals are in a highly susceptible group, as their host defense is indigent; their defenses are already down, which could result in an increasingly poor prognosis for them [182]. Atmospheric temperature variation also plays a role in the spread of COVID-19. It is known that winter is the flu season in cold temperate countries because influenza viruses survive better in cold-dry weather with reduced ultraviolet light. Reduced innate immunity is due to low levels of vitamin D and melatonin and to staying indoors frequently during winter, increasing opportunities for the virus to spread among people [185, 186]. COVID-19 started during winter, and it has been shown that the local immune response in the nasal passage is reduced in people during cold winter, making them more susceptible to flu-like viruses [186]. This situation may make people living in such areas more vulnerable than those living in tropical areas, leading to differences in the spatial distribution of COVID-19 worldwide.

High temperature may be a protective factor, as it can provoke the evaporation of respiratory droplets, reducing infectivity [187–189]. African and Asian tropical countries where high temperatures and high relative humidity are common may be protected from COVID-19 because of their negative effect on the spread of the disease. On the other hand, in Western countries where there are low temperatures and high humidity, its transmission in air-conditioned and temperate climatic environments may be facilitated [187, 189]. Closed areas with low airflow and ventilation have been shown to play a role in the increasing risk of COVID-19 infection [162], which makes developed countries at greater risk than developing countries. For instance, in developing countries, apart from some places in large cities, most people live under more natural atmospheric temperatures than do people in Western countries, where most houses are built with air-conditioning systems. Living in air-conditioned housing in addition to other factors, such as being older, having respiratory diseases or diabetes, or being an immune-compromised person, may increase the risk of transmission of COVID-19 [190–193].

High population density has also been associated with the spread of epidemics, including COVID-19 [194]. In the case of COVID-19, a high number of cases have been observed in overcrowded cities in countries such as China, Japan, India, and the United States. Additionally, countries with a high proportion of elderly populations, such as Italy and Spain, have a higher risk of severity [181], and this risk increases when young and older people live together [195].

International travel and trade have facilitated the spread of COVID-19. In the past, humanity has survived previous pandemics by infectious agents such as influenza viruses. However, the present one is unprecedented in its capacity to take advantage of modern globalization, allowing for the massive transborder spread at a surprising speed [196]. With the current international travel and trade, it has been quite difficult to contain the spread of this virus. Countries have tried to impose travel restrictions and have reduced the exportation rate from mainland China, but these restrictions are insufficient to contain the geographic spread. Such measures have led to discrimination against foreigners [197]. However, travel restrictions cannot be expected to fully apprehend the global expansion of COVID-19 but may decrease the rate of case exports if enacted during the early stages of the epidemic. This is because the onset of symptoms at the time of arrival is, in most cases, not detectable as the virus is still under incubation period [198].

Delayed government response, inadequate infrastructures, and lack of biosafety and biosecurity awareness have also contributed to the transmission and spread of COVID-19. Most countries have facilitated the transmission and spread of the disease due to inadequate preventive and/or mitigating strategies. Many countries failed to prevent the epidemic in the beginning by delaying preventive measures. However, a few countries succeeded in controlling the outbreak by adopting an effective mitigating strategy. South Korea and other countries, such as Singapore, Hong Kong, Japan, and Germany, quickly responded after the first case by adopting a strategy involving the testing and isolation of cases, contact tracing, and quarantining of suspected cases to suppress the full transmission of the virus in communities [199]. Additionally, countries that have experienced SARS-like epidemics such as Taiwan, Hong Kong, and South Korea have successfully prevented the spread of COVID-19 from China by adopting preventive measures such as travel bans, closing of borders to nonresidents, and 14-day quarantines [200]. Regarding lockdown measures, in the beginning, some countries delayed adopting this strategy because they sought to prevent harm to their economy (including some developed and developing countries) and because of poverty, especially in developing countries, where people are forced to go out as they need to work for their daily livelihood.

Inadequate infrastructures and equipment and lack of awareness regarding laboratory biosafety and biosecurity in certain countries, especially in developing countries, have contributed to the spread of the disease in hospitals. Handling pathogens, especially viruses, may expose health workers to a high risk of contamination if they are not well trained. Thus, to prevent nosocomial infections, sufficient training of health workers in the principles and procedures of good laboratory practice (GLP) is needed. Health workers should be well trained on biohazards and the safe management of biological agents in the laboratory. Laboratory workers must have sufficient knowledge and experience about how to handle emerging pathogens safely and securely [201, 202]. However, good awareness of GLPs must be reinforced by the availability of adequate equipment and infrastructures. In many countries, health professionals are working without PPE and are exposed to the infection. Thus, the lack of awareness about COVID-19 preventive measures cause health workers to be exposed and become infected. Additionally, the lack of testing kits, ventilators, and other equipment is impeding some countries from adopting an effective testing and tracing strategy and preventing deaths among severe cases.

## Conclusions

Human activities are still the significant determinants of the transmission and spread of (re)emerging infectious diseases. To date, we are still dealing with the COVID-19 pandemic, and it is challenging to predict when it will end. However, it is possible to face the disease or to reduce the risk of its transmission and spread by changing human behavior and implementing better early warning systems and effective disease control and prevention. Thus, effective global surveillance, global political will, and multidisciplinary collaboration of all stakeholders will be helpful in responding to any future threats.

## Data Availability

Not applicable.

## Abbreviations

AIDS: Acquired immunodeficiency syndrome
AMR: Antimicrobial resistance
CA-MRSA: Community-associated methicillin-resistant Staphylococcus aureus
CDC: Centers for Disease Control and Prevention
COVID-19: Coronavirus disease 2019
CTX-M: Cefotaxime-M
DRC: The Democratic Republic of the Congo
EIDs: Emerging infectious diseases
EVD: Ebola virus disease
FWBDs: Food- and water-borne diseases
HIV: Human immunodeficiency virus
MARS: Middle East respiratory syndrome
MDR: Multidrug-resistant
MRSA: Methicillin-resistant *Staphylococcus aureus*
SARS: Severe acute respiratory syndrome
SDGs: Sustainable Development Goals
TB: Tuberculosis
EU: European Union
UK: United Kingdom of Great Britain and Northern Ireland
UNHCR: The Office of the United Nations High Commissioner for Refugees
US: The United States
VBDs: Vector-borne diseases
WHO: World Health Organization
